# The Role of Pattern-Recognition Receptors in Graft-Versus-Host Disease and Graft-Versus-Leukemia after Allogeneic Stem Cell Transplantation

**DOI:** 10.3389/fimmu.2014.00337

**Published:** 2014-07-18

**Authors:** Simon Heidegger, Marcel R. M. van den Brink, Tobias Haas, Hendrik Poeck

**Affiliations:** ^1^III. Medizinische Klinik, Klinikum Rechts der Isar, Technische Universität München, Munich, Germany; ^2^Department of Medicine and Immunology, Memorial Sloan-Kettering Cancer Center, New York, NY, USA

**Keywords:** graft-versus-host disease, allogenic hematopoietic stem cell transplantation, pattern-recognition receptors, inflammsome, microbiota, danger molecules

## Abstract

Allogeneic hematopoietic stem cell transplantation (allo-HSCT) is the only treatment with curative potential for certain aggressive hematopoietic malignancies. Its success is limited by acute graft-versus-host disease (GVHD), a life-threatening complication that occurs when allo-reactive donor T cells attack recipient organs. There is growing evidence that microbes and innate pattern-recognition receptors (PRRs) such as toll-like receptors (TLR) and nod-like receptors (NLR) are critically involved in the pathogenesis of acute GVHD. Currently, a widely accepted model postulates that intensive chemotherapy and/or total-body irradiation during pre-transplant conditioning results in tissue damage and a loss of epithelial barrier function. Subsequent translocation of bacterial components as well as release of endogenous danger molecules stimulate PRRs of host antigen-presenting cells to trigger the production of pro-inflammatory cytokines (cytokine storm) that modulate T cell allo-reactivity against host tissues, but eventually also the beneficial graft-versus-leukemia (GVL) effect. Given the limitations of existing immunosuppressive therapies, a better understanding of the molecular mechanisms that govern GVHD versus GVL is urgently needed. This may ultimately allow to design modulators, which protect from GvHD but preserve donor T-cell attack on hematologic malignancies. Here, we will briefly summarize current knowledge about the role of innate immunity in the pathogenesis of GVHD and GVL following allo-HSCT.

## Introduction

Allo-HSCT is an established treatment modality for aggressive hematological malignancies and is performed in more than 30,000 patients annually worldwide (1). Donor-derived T cells in the graft can maintain remission after induction therapy by attacking residual tumor cells in a process known as graft-versus-leukemia (GVL). Unfortunately, beneficial GVL effects are tightly associated with the pathogenesis of acute graft-versus-host disease (GVHD). Allogeneic donor T cells recognize mismatches in major or minor histocompatibility antigens present in non-malignant host tissues and subsequently induce immune-mediated damage to target organs such as the gastrointestinal tract, skin, liver, and lungs (2). Acute GVHD occurs in 40–50% of all allo-HSCT patients and accounts for considerable morbidity and mortality (3). Depletion of T cells from the allograft can decrease the incidence of acute GVHD, but comes at the cost of greater risk of graft failure, reduced GVL activity, and increased incidence of leukemic relapse (4). As a current standard of care for GVHD, glucocorticoids and other immunosuppressive drugs are used to inhibit T-cell activation and proliferation, which similarly affects GVL activity. A better understanding of the underlying molecular mechanisms may help to design measures to prevent GVHD but preserve donor T-cell responses and GVL activity, thus allowing for a broader application of allo-HSCT in the future. Here, we discuss how the innate immune system and its environmental triggers shape the clinical course and outcome of allo-HSCT in patients and corresponding animal models.

The biology and function of pattern-recognition receptors (PRRs) is reviewed in detail within this research topic issue (5, 6). In brief, PRRs are germ line-encoded receptors that detect conserved molecular structures that are specific to invading microbes but are absent on host cells under homeostatic conditions. Ligation of such pathogen-associated molecular patterns (PAMPs) leads to activation and maturation of antigen-presenting cells (APCs), release of pro-inflammatory cytokines and, eventually, the initiation of an adaptive immune response. PRRs are expressed on different cell types of the innate and adaptive immune systems as well as non-hematopoietic cells such as endo- and epithelial cells.

## Importance of Host Microbiota and the Emerging Role of Innate Immunity in GVHD

Primary target organs of acute GVHD such as the gastrointestinal tract, skin, liver, and lungs all form epithelial linings that constantly interact with commensal and pathogenic bacteria, either through the epidermis, intestinal, or airway mucosa or the portal circulation. Consistently, there is growing evidence that bacteria and innate PRRs are critically involved in the pathogenesis of acute GVHD. Landmark studies by van Bekkum and colleagues in mice demonstrated that bacterial decontamination or utilization of germ-free mice lead to less severe intestinal GVHD (7, 8). Reduction of intestinal microbiota by antibiotic treatment not only mitigated intestinal but also skin GVHD, suggesting a systemic effect of gut decontamination (9). Similarly, antibiotic decontamination in patients undergoing allo-HSCT seemed to confer robust protection from acute GVHD (10, 11). Lipopolysaccharide (LPS) derived from Gram-negative bacteria was identified as a driver of GVHD pathogenesis. In experimental models, allo-HSCT recipients that were treated either with anti-endotoxin neutralizing antibodies (12, 13) or an oral LPS inhibitor (14) showed reduced GVHD severity associated with preserved GVL effects and improved overall survival. These findings launched widespread use of prophylactic antibiotic treatment to reduce the bacterial burden prior to allo-HSCT, now routinely performed in many transplantation centers worldwide (15). Interestingly, modification of the intestinal microbiota using the probiotic microorganism *Lactobacillus rhamnosus* resulted in reduced translocation of enteric bacteria to the mesenteric lymph nodes, associated with improved survival and reduced acute GVHD in mice (16). Furthermore, intestinal inflammation during GVHD in mice and humans is associated with major shifts in the composition of the intestinal microbiota. In one report, GVHD-associated loss of paneth cells resulted in reduced production of antimicrobial peptides and a loss of microbial diversity with outgrowth of *Escherichia coli*. Antibiotic treatment prevented outgrowth of *E. coli* and ameliorated the course of GVHD (17). Another study showed a marked expansion of *Lactobacillales* in murine GVHD. Elimination of this species from the flora of mice before allo-HSCT aggravated GVHD, whereas its reintroduction mediated significant protection, indicating that the microbiota can modulate the severity of intestinal inflammation (18). A recent study suggested that not only bacteria but also host fungal communities (mycobiome) can critically shape acute GVHD (19). Patients colonized with candida species suffered from more severe GVHD and showed more frequent intestinal involvement (33 versus 19%). Interestingly, candida colonization was more frequent in patients bearing a loss-of-function single nucleotide polymorphism (SNP) that is associated with impaired function of the innate PRR Dectin-1, a member of the C-type lectin family of receptors that detect carbohydrates constituent of fungal cell walls, thus playing an important role in the initiation of antifungal immunity (20).

**Figure 1:**
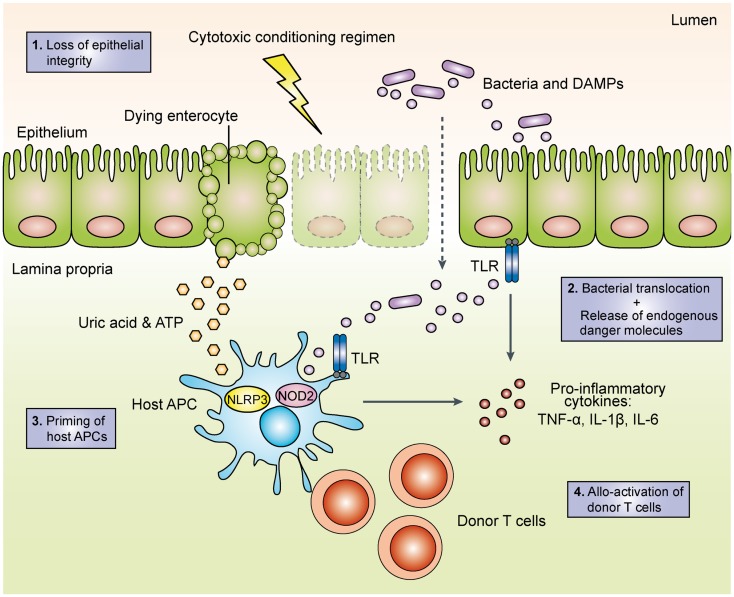
**Schematic overview of the initiation phase of acute graft-versus-host disease**. During the toxic conditioning regimen with total-body irradiation and/or chemotherapy, the destruction of intestinal epithelial cells leads to the loss of the epithelial barrier function. The subsequent translocation of luminal bacteria as well as the release of endogenous danger molecules such as adenosine triphosphate (ATP) and uric acid result in the production of pro-inflammatory cytokines. Activated host and/or donor antigen-presenting cells then prime allo-reactive donor T cells, which perpetuate acute GVHD. TLR, toll-like receptor; APC, antigen-presenting cell; DAMP, danger-associated molecular pattern; TNF, tumor necrocis factor; IL, interleukin; NOD2, nucleotide-binding oligomerization domain; NLRP3, NACHT, LRR, and PYD domains-containing protein 3.

With increasing knowledge on how PRRs detect conserved microbial and danger-associated molecular patterns (DAMPs) and initiate adaptive immune responses, their role in the pathogenesis of acute GVHD has become a focus of intense research. A widely accepted model (depicted in Figure 1) postulates that intensive chemotherapy and/or total-body irradiation (TBI) during pre-transplant conditioning results in tissue damage and loss of epithelial barrier function. Bacterial components translocated across the barrier as well as endogenous danger molecules released from damaged cells are sensed by PRRs on host and/or donor APCs such as dendritic cells (DCs), which produce pro-inflammatory cytokines and prime allo-reactive donor-derived T cells (21). This model is supported by mouse studies, which demonstrate that intensified TBI increases epithelial damage and is associated with more severe GVHD (14, 22). Intriguingly, innate lymphoid cell-derived IL-22 protects both the intestinal stem cell compartment and the mature intestinal epithelium from inflammatory tissue damage (23) in line with the general concept that IL-22 can maintain epithelial integrity under inflammatory conditions (24). The enhanced intestinal barrier function thus may limit LPS translocation and subsequent PRR activation. Consistently, genetic deficiency for IL-22 results in impaired gut epithelial integrity and increased tissue damage and mortality from acute GVHD (23). Along these lines, prophylactic treatment with recombinant keratinocyte growth factor protected mice from the development of lethal acute GVHD, presumably via reduction of intestinal epithelial apoptosis and diminished LPS-mediated pro-inflammatory cytokine release (25). However, administration of the recombinant human keratinocyte growth factor palifermin before and after allo-HSCT in a phase I/II placebo-controlled clinical trial had no significant effect on the incidence and severity of acute GVHD and short-term survival (26), presumably due to pleiotropic effects of palifermin.

## Toll-Like Receptors in GVHD Pathogenesis

Toll-like receptors (TLRs) constitute a family of transmembrane PRRs that are broadly expressed in hematopoietic and non-hematopoietic cells (27). TLR ligation by a variety of microbial components leads to activation of APCs, production of pro-inflammatory cytokines, and release of chemokines. One of the best-studied TLRs in the context of GVHD is TLR4, which detects LPS in the cell wall of Gram-negative bacteria. The importance of LPS translocation and subsequent release of pro-inflammatory cytokines such as TNF-α for the pathogenesis of acute GVHD have been clearly documented (14). Moreover, genetic deficiency for TLR4 in either donor or recipient cells resulted in reduced DC activation, dampened allogenic T-cell proliferation, and less severe acute GVHD (28). However, signaling through TLR4 seems not to be absolutely required for the development of GVHD in all cases. Accordingly, in another study TLR4-deficient recipient mice showed GVHD severity comparable to wild-type mice (29), suggesting that alternative pathways in the absence of TLR4 signaling can lead to the activation of host APCs and subsequent donor T-cell stimulation. Genetic association studies in patients undergoing allo-HSCT have shown inconsistent results concerning the role of TLR4 in the pathogenesis of GVHD. Patients showed reduced frequency of severe GVHD when they or their sibling donors carried at least one of two SNPs that are associated with reduced TLR4 responsiveness to LPS (odds ratio of 0.63 and 0.88, respectively) (30). A second study showed that if both patient and donor carry the SNP Thr399Ile, the incidence of severe acute GVHD was significantly increased but overall survival was not influenced (31). These contrasting results may be attributable to differences in patient cohorts, conditioning regimens and antimicrobial treatment routines.

Other members of the TLR family have been associated with immunomodulatory capacities and suppression of GVHD. Pretreatment of mice with the TLR5 ligand flagellin resulted in reduced GVHD and improved overall survival (32). Interestingly, in a clinical study of adoptively transferred immunosuppressive regulatory T cells to allo-HSCT recipients, patients who developed GVHD showed significantly increased TLR5 mRNA expression in peripheral blood mononuclear cells (33), whereas patients that did not show GVHD had reduced TLR5 mRNA expression. These results in the human system are difficult to interpret but may indirectly suggest a pro-inflammatory role of TLR5 in allo-HSCT recipients, contrary to the mouse study cited above.

Furthermore, it was shown that tissue inflammation induced by TLR ligation can modulate the development of GVHD at a local level (34). In this regard, the authors created mixed chimeras by transplanting B6 bone marrow cells into lethally irradiated BALB/c mice. After establishment of the B6 allograft, they transferred additional B6 donor T cells, which mimic the clinical use of donor lymphocyte infusions. Transplantation of donor T cells into established mixed chimeras did not induce GVHD, as donor T cells did not enter target tissues despite undergoing allo-activation, expansion, and up-regulation of homing molecules. Strikingly, topical application of R-848, a synthetic TLR7 agonist, unleashed massive skin infiltration of donor T cells, and development of localized GVHD. Using a different TLR7 ligand (3M-011), another group demonstrated that the timing of TLR activation has important consequences for the pathogenesis of GVHD. While repetitive applications of 3M-011 after allo-HSCT aggravated GVHD severity (35), a single treatment timed between TBI and allo-HSCT induced expression of the immunoinhibitory enzyme indoleamine 2,3-dioxygenase (IDO) in host APCs, which resulted in reduced lethal intestinal GVHD (36).

In addition, signaling via TLR9 that detects microbial CpG-DNA motifs has been implicated in the pathogenesis of acute GVHD. Studies in TLR9 deficient mice showed reduced GVHD and improved survival (29, 37). Repetitive application of CpG-DNA following allo-HSCT results in increased GVHD mortality (35). This effect was dependent on TLR9 signaling and subsequent IFN-γ release in host hematopoietic cells. Less consistent results come from human studies: Transplant patients who carry gene variants associated with reduced TLR9 expression showed GVDH occurrence similar to control patients (38). A recent report analyzed two alternative SNPs that have been described to interfere with the TLR signaling pathway (39). While patients receiving stem cells from an unrelated donor with the A1174G variant experienced severe acute GVHD more frequently (49.5 vs. 20.7%), the T1635C variant in donor cells was associated with protective effect against severe acute GVHD (16.7 vs. 49.1%).

Taken together, TLR signaling can both aggravate and attenuate the development of local and systemic GVHD; critical factors seem to be the cell type primarily affected (e.g., hematopoietic versus non-hematopoietic) and the time point of TLR ligation. Thus, the role of TLRs in the pathophysiology of GVHD remains controversial. Recipient mice that are genetically deficient for either the TLR signaling adaptor molecules MyD88 or TRIF were found to show less severe intestinal GVHD (37). In a contrasting report, bone marrow chimeric recipient mice deficient for MyD88 and/or TRIF only in hematopoietic cells developed GVHD comparable to wild-type controls (40). Other than by differences in the experimental setting between institutions (e.g., microbiota and conditioning regime), these differences might be explained by alternative (non-TLR) pathways in APCs or epithelial cells, leading to allo-activation and proliferation of donor T cells in the absence of TLR signaling.

## Nod-Like Receptors in GVHD

Another family of PRRs with relevance to GVHD is the cytoplasmic NOD-like receptors (NLRs). NOD1 and NOD2 detect peptidoglycans as components of the bacterial cell wall (6). Both receptors have been extensively studied in the context of Crohn’s disease, a chronic inflammatory bowel disease that shares several immunopathogenic features with intestinal GVHD. Reduced NOD2 activity was found to be associated with impaired epithelial barrier function and aggravated intestinal inflammation (41). Similarly, following allo-HSCT, *NOD2*-deficient mice showed signs of exacerbated GVHD (42). Another study with bone marrow chimeric mice that lacked NOD2 activity only in hematopoietic cells showed that NOD2 negatively regulates the development of GVHD through its inhibitory effect on host APCs. The presence of different SNPs in the *NOD2* coding region resulting in impaired downstream signaling via the pro-inflammatory transcription factor NF-κB in either the patient, donor or both was associated with more severe GVHD (43). Two follow-up reports confirmed *NOD2* mutations as independent risk factor for transplant-related mortality (44, 45). However, several studies proposed contrasting data as they could not find an impact of *NOD2* polymorphisms on GVHD severity and outcome after allo-HSCT (46–48).

Several members of the NLR family not only detect microbial invaders but also survey cellular homeostasis and sense endogenous danger signals (6). Examples of such DAMPs are adenosine triphosphate (ATP), uric acid crystals, and double-stranded DNA released from dying cells. Activation of specific members of the NLR family by DAMPs results in the formation of cytosolic multi-protein complexes called inflammasomes, whose exact composition depends on the activator initiating their assembly (49). Inflammasome activation leads to the cleavage of pro-caspase-1 and the subsequent processing of the bioactive form of IL-1β and IL-18. These downstream effector molecules have been shown to modulate GVHD as antibody-mediated neutralization of IL-1β resulted in less severe acute GVHD in mice (50, 51). In a phase I/II clinical trial, blockade of IL-1 signaling attenuated GVHD in 8 out of 14 patients with glucocorticoid-refractory disease (52). In contrast, a larger randomized study showed no effect of a recombinant IL-1 receptor antagonist on GVHD severity and overall survival (53). However, timing and way of administration of IL-1 receptor blockade may be critical. Novel IL-1β specific antibodies await clinical testing in the setting of allo-HSCT.

The NLRP3-inflammasome is an essential platform for caspase-1 activation in response to multiple distinct exogenous and endogenous danger signals (6) and its function can be regarded as a guardian of intracellular homeostasis. NLRP3 utilizes the adapter protein ASC for activation of caspase-1 and subsequent cleavage of the precursor protein pro-IL-1β into its active form. Binding of the endogenous danger molecule ATP to the purinergic receptor P2X_7_ leads to potassium efflux and subsequent activation of the NLRP3-inflammasome. In mice and humans undergoing allo-HSCT, increased extracellular levels of ATP were found after TBI and during the development of GVHD (54). ATP released from damaged or dying cells induces activation of host APCs and priming of allo-reactive donor T cells. Pharmacological metabolization of ATP using apyrase resulted in less severe GVHD (54). Chimeric mice that were genetically deficient for the purinoceptor *P2X*_7_ in hematopoietic cells were partially protected from GVHD. Reconstitution with wild-type DCs resulted in restored GVHD development, demonstrating a critical role for host DCs in sensing ATP and the subsequent induction of GVHD. However, significantly reduced overall survival but no alterations in GVHD severity were found in patients or corresponding donors with a loss-of-function SNP in the *P2X_7_* *receptor* gene (55). After conditioning therapy in mice, intestinal commensal bacteria and uric acid contribute to NLRP3-inflammasome-mediated IL-1β processing, and gastrointestinal decontamination or enzymatic uric acid depletion led to reduced GVHD severity (51). NLRP3 and the adapter protein ASC, which are both required for pro-IL-1β cleavage, were critical for the full manifestation of GVHD. In transplanted mice, IL-1β exerted its effects on both DCs and T cells, which preferably differentiated into IL-17A-producing Th17 cells (51), a CD4^+^ T-cell subpopulation that has been causally linked to instances of aggravated GVHD after allo-HSCT (56). Donors carrying one of two genetic alterations in the non-coding regions of the *NLRP3* gene are associated with increased disease relapse and reduced overall survival but no alterations in GVHD severity in allo-HSCT patients (57). Thus, directed therapies targeting the NLRP3-inflammasome or depletion of specific DAMPs remain promising therapeutic options to reduce the level of systemic inflammation in the setting of allo-HSCT, but data reported so far are somewhat controversial and await further clarification.

In summary, NOD2 signaling in hematopoietic cells appears to protect from acute GVHD. Conflicting data from genetic association studies in humans are most likely attributable to differences in frequency of *NOD2* SNPs between patient cohorts, and differences with conditioning, immune suppression, and antibiotic protocols (44). We refer to Ref. (58) for a more detailed discussion of NOD2 in GVHD. Data on inflammasomes in allo-HSCT are not yet abundant, but NLRP3 and possibly other inflammasomes that sense endogenous danger signals such as ATP and uric acid and induce IL-1β release seems to have a role in the pathogenesis of acute GVHD.

## Innate Pattern-Recognition Receptors and the Graft-Versus-Leukemia Effect

Many studies have highlighted the fact that innate PRRs contribute to the inflammatory processes that lead to activation of allo-reactive T cells and the pathogenesis of GVHD. In contrast, the molecular details that shape the beneficial GVL effect remain poorly understood. Yet, only a detailed molecular understanding of the GVL effect will allow for the discrimination between GVL-pathways and allo-immune reactions that drive clinical GVHD, a prerequisite for broader application of allo-HSCT in the future. Unspecific depletion or proliferative inhibition of donor T cells is believed to come at the cost of increased relapse of the underlying malignant disease (59). However, recent data challenge that view, since T-cell depletion via selection of CD34^+^ cells in the allograft was found to be associated with markedly reduced GVHD but no differences in the rate of leukemic relapse (60, 61). Yet, data on the role of PRRs in GVL remain scarce. Studies that showed an association between loss-of-function SNPs in the *NOD2* gene and the severity of GVHD found no impact on the relapse rate by these same mutations (43, 45). Thus, NOD2 would seem to be an attractive pharmacological target to attenuate GVHD without interfering with the GVL effect. However, other studies that investigated the same *NOD2* SNPs in transplant patients and corresponding donors could not confirm their effect on GVHD pathogenesis (52), or showed an increased risk of relapse and death if recipients and/or donors were carrying such an alteration in the *NOD2* gene (62, 63). These contrasting results emphasize that data on differential regulation of GVHD versus GVL by PRRs on a systemic level are still premature and do not yet allow for systemic modulation of PRRs as a general treatment approach. In contrast, as PRRs can control the development of GVHD at a local level (34), their pharmacological manipulation in specific immune compartments seems to be a more promising approach. Interfering with PRR signaling in GVHD target tissues, such as intestine and skin, but sparing lymphoid organs and bone marrow, where residual hematologic malignancies reside, may allow to efficiently target GVHD but leaving GVL intact.

## Conclusion and Future Directions

Toll-like receptors and NLRs respond to a variety of microbial and endogenous danger signals and there is increasing evidence that they influence the development of acute GVHD. Yet, the role of TLRs in the pathophysiology of GVHD remains controversial, as studies with TLR4- and MyD88-deficient mice demonstrated that TLR signaling may not be absolutely required for the development of GVHD. Loss-of-function mutations in the *NOD2* gene, on the other hand, correlated in some studies with adverse allo-HSCT outcome in humans, suggesting a protective role of NOD2. Furthermore, activation of the NLRP3-inflammasome during early conditioning in mice contributes to the development of acute GVHD. Other receptors involved in the local control of microbiota will be the focus of future studies. Type I interferon has been shown to play an important role in defining the balance between GVHD and GVL responses (64). Thus, PRRs that detect cytosolic nucleic acids and lead to the production of large amounts of type I interferon such as the family of RIG-I-like helicases (5) or the recently discovered cytosolic DNA receptor cyclic GAMP synthase (cGAS) and its adapter STING (65) are of particular interest. Unraveling their role in acute GVHD will not only boost our understanding of this major complication after allo-HSCT, but may allow for novel therapeutic approaches to GVHD and related disorders like inflammatory bowel disease.

In light of the contradicting data regarding the role of some PRRs in acute GVHD, we would like to point out some of the major obstacles in the field of allo-HSCT research. Mouse models of GVHD are heterogeneous, with different subsets of immune cells being the main drivers of respective GVHD pathologies. In addition, innate and adaptive immunity are influenced by intestinal microbiota, which can vary critically between different breeding facilities. The effect of a given genetic alteration or therapeutic intervention may therefore differ between models and breeding facilities, and interpretation of such data must be undertaken with caution. Parts of the existing data may have to be revised in light of these new perceptions. Awareness of these difficulties together with increasing knowledge of graft and host immune and microbial physiology will, however, make this task easier in the future.

## Conflict of Interest Statement

The authors declare that the research was conducted in the absence of any commercial or financial relationships that could be construed as a potential conflict of interest.
